# Early loss of immunity against measles following allogeneic hematopoietic stem cell transplantation

**DOI:** 10.1002/ajh.25590

**Published:** 2019-08-16

**Authors:** Hannah M. Garcia Garrido, Mariëlle van Aalst, Janke Schinkel, Gerrit Koen, Jacqueline M. Defoer, Mette D. Hazenberg, Erfan Nur, Martin P. Grobusch, Sascha S. Zeerleder, Abraham Goorhuis, Godelieve J. de Bree

**Affiliations:** ^1^ Department of Infectious Diseases Amsterdam UMC‐location AMC, The Amsterdam Infection and Immunity Institute (AI&II), University of Amsterdam Amsterdam The Netherlands; ^2^ Center for Tropical Medicine and Travel Medicine, Amsterdam UMC‐Location AMC The Amsterdam Infection & Immunity Institute (AI&II), University of Amsterdam Amsterdam The Netherlands; ^3^ Department of Medical Microbiology, Clinical Virology Amsterdam UMC‐location AMC, University of Amsterdam Amsterdam The Netherlands; ^4^ Department of Hematology Amsterdam UMC‐location AMC, University of Amsterdam, and Cancer Center Amsterdam, University of Amsterdam Amsterdam The Netherlands; ^5^ Department of Immunopathology, Sanquin Research Amsterdam UMC‐location AMC, The Amsterdam Infection & Immunity Institute (AI&II), University of Amsterdam Amsterdam The Netherlands; ^6^ Department of Hematology and Central Hematology Laboratory Inselspital, Bern University Hospital and Department for BioMedical Research, University of Bern Bern Switzerland; ^7^ Department for BioMedical Research University of Bern Bern Switzerland; ^8^ Amsterdam Institute for Global Health and Development Amsterdam The Netherlands

To the Editor:

1

Measles is a potentially life‐threatening illness in immunocompromised patients.^1^ The waning measles vaccine coverage ‐ resulting in recent measles outbreaks ‐ puts these vulnerable patients at incremental risk.^2^ Allogenic hematopoietic stem cell transplantation (allo‐HSCT) recipients constitute an exceptionally vulnerable subgroup, and it has been demonstrated that a significant proportion of these patients loses protective immunity against measles.^3^, ^4^ The time course for this loss of immunity after allo‐HSCT is not known; in addition, patients at particular higher risk to losing measles immunity cannot be identified. Vaccination with the life‐attenuated measles vaccine is considered safe at least 2 years after allogeneic HSCT, and only in the absence of graft vs host disease (GvHD). In addition, previous studies mainly assessed patients who received myeloablative conditioning (MAC) regimens, while reduced intensity conditioning (RIC) regimens are currently more common, and may have less impact on loss of measles immunity. The aims of this study were to assess immunity to measles during the 1 year after MAC or RIC allo‐HSCT, and to identify patient or treatment related factors that may predispose to loss of measles immunity after allo‐HSCT.

We included all patients who underwent allo‐HSCT at the Amsterdam UMC, the Netherlands, between 2010 and 2017, if they had available serum or plasma samples before allo‐HSCT, and at 3 months and/or at 1‐year after allo‐HSCT. During this period, allo‐HSCT was performed at our institute with peripheral blood stem cell grafts, from HLA‐identical matched sibling donors (MSD) or matched‐unrelated donors (MUD). Prophylaxis for graft‐vs‐host disease (GvHD) consisted of a combination of cyclosporine A/tacrolimus, and mycophenolate mofetil. Childhood measles vaccination has been implemented in Dutch vaccination guidelines for infants born from 1976, and coverage in the Netherlands has been stable around 95% until recently.^2^ Therefore, we assumed that patients with detectable measles IgG before allo‐HSCT, born before 1976, had acquired immunity through natural exposure. Patients born after 1976 were considered to have vaccine induced measles immunity.

Measles‐specific IgG levels were determined before allo‐HSCT, and at 3 months and/or 1 year after allo‐HSCT, using Liaison XL (DiaSorin) chemiluminescene (CLIA) technology. Samples were analyzed in batch, and measles protective immunity was defined as an IgG level above 120 mIU/mL.^5^

The primary outcome was the proportion of patients with protective immunity to measles at 3 months and 1 year after allo‐HSCT. Secondary outcomes were changes in measles‐specific IgG values and potential factors associated with immunity loss (sex, age, natural or vaccine‐induced immunity, conditioning regimen, chimerism, type of donor, presence of GvHD). Immunity loss was defined as a measles IgG value below 120 mIU/mL at three or 12 months after allo‐HSCT, among patients who had protective IgG values at baseline. We used a generalized linear mixed model with fixed effects for the dichotomous outcome, the Wilcoxon signed rank test for changes over time (paired analysis), and logistic regression the two‐sided Mann‐Whitney *U* test differences between groups. All analyses were performed with IBM SPSS version 23.0.

Between 2010 and 2017, 256 patients received an allo‐HSCT at the Amsterdam UMC‐AMC. Of these patients, 91 had available serum samples before allo‐HSCT, and at 3 months (n = 78) and/or 12 months (n = 84) after allo‐HSCT, and were included in the study. Median age was 54 years (IQR 41‐61). The most common indication for allo‐HSCT was acute myeloid leukemia (54%). Of all patients 85% received RIC. Grade II‐IV acute GvHD was present in 51% of all patients, and by the end of the study period 35% had developed moderate or severe chronic GvHD. In 79% of the patients, they had naturally acquired measles immunity before transplantation. Only two patients used intravenous or subcutaneous immunoglobulins during the study period. Baseline characteristics are provided in Table S1.

Measles immunity significantly declined from 91% before allo‐ HSCT (85/91 patients) to 86% (67/78 patients), at 3 months after allo‐HSCT, and 61% (55/84 patients) at 1 year after allo‐HSCT (*P*‐value). Median IgG levels decreased in all patients, but this was most apparent for patients who had received MAC (Figure 1A) and patients with vaccine induced immunity (Figure 1B), due to lower pre‐transplantation antibody levels.

**Figure 1:**
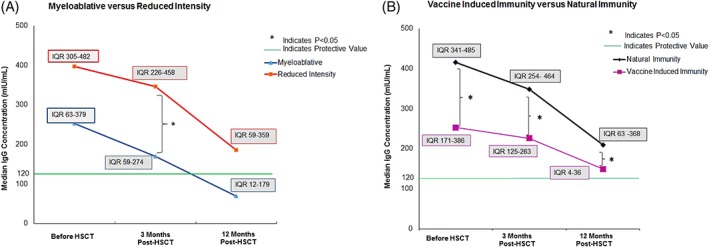
Decline in measles‐specific IgG levels before and after allogeneic HSCT in A, MAC vs RIC patients and B, patients with vaccine vs naturally acquired measles immunity. Dots indicate the median IgG level. *The Mann Withney *U* test was used to test for statistical significance between groups. IQR, Interquartile range

Patients with a MAC regimen had a higher risk of immunity loss compared to RIC patients, 3 months after allo‐HSCT (36% vs 6.8% respectively; Odds Ratio 0.12; 95% CI 0.02‐0.58). This association remained statistically significant when adjusting for age, vaccine/naturally acquired immunity, and GvHD (data not shown). When measured 1 year after allo‐HSCT, the difference between MAC and RIC patients was no longer significant, with 55% and 31% immunity loss in MAC and RIC patients, respectively (OR 0.38, CI 0.10‐1.39). We did not find statistically significant associations between loss of measles immunity and other studied factors, such as sex, age, donor type, vaccine/natural pre‐HSCT measles immunity, acute/chronic GvHD (Table S2).

We observed measles seroconversion from negative to positive in two patients. One patient tested negative at baseline (31 mIU/mL), positive 3 months after allo‐HSCT (358 mIU/mL), and again negative at 1 year after allo‐HSCT (14 mIU/mL). Another patient tested positive at baseline (188 mIU/mL), negative at 3 months (60 mIU/mL), and positive again 1 year after allo‐HSCT (151 mIU/mL). These patients had not received immunoglobulins, nor had they been vaccinated during the study period. There had been no clinical signs of measles in these patients.

We show here that a large proportion of patients becomes vulnerable to measles in the first year after allo‐HSCT, due to waning measles‐specific IgG levels below the limit of protection. Our results add to older studies showing loss of measles immunity at later time points (from 2 years onwards) after allo‐HSCT, predominantly in patients who had received MAC.^3^, ^4^ It is assumed that in (newer) RIC as opposed to MAC regimens, host antibody producing plasma cells may survive longer, or may not be replaced at all, which may explain longer persistence of measles immunity.^3^ At 1 year post transplantation however, we found a striking immunity loss in both MAC and RIC patient groups.

Natural measles infection is known to induce higher antibody levels compared to vaccination.^3^ In our study, RIC patients more often had naturally acquired measles immunity compared to MAC patients, (92% vs 46%, *P* < .01), resulting in higher pre‐allo HSCT median measles IgG levels. Nevertheless, a similar strong decline of measles IgG levels was observed regardless of conditioning regimen and type of previous immunity.

Interestingly, two patients experienced an increase in measles specific antibody titers during the 1 year after allo‐HSCT. Since these patients had not received IVIG, nor had been vaccinated against measles, the most likely explanation was a passive transfer of measles IgG by repeated thrombocyte transfusions, which both patients had received during the study period.

The re‐occurrence of large‐scale measles epidemics is a worrying development, and is particularly dangerous for allo‐HSCT recipients, who lose measles immunity after transplantation. Our study indicates that this also applies to patients treated with a RIC regimen, long before they become eligible for (re‐)vaccination. Given the increasing risk of exposure to measles, we would advise assessment of measles IgG levels at regular time intervals, when patients are planning to travel to measles endemic countries, or in case of local outbreaks. Strategies to prevent measles in seronegative allo‐HSCT recipients include giving immunoglobulins (passive immunization), and vaccination (active immunization). The first is usually applied to protect contacts of measles patients, when vaccination is considered dangerous. Although vaccination is generally not recommended before at least 2 years after allo‐HSCT, without active GvHD or use of immunosuppressive agents, early measles vaccination (eg, 1 year after allo‐HSCT) has been performed in an outbreak setting, and has been shown to be safe and effective.^6^ In an emergency setting, the approach of early vaccination could therefore be considered.

## CONFLICT OF INTEREST

None of the authors have any competing interests to declare. No funding received.

## Supporting information


**Table S1** Baseline Characteristics
**Table S2**: Factors associated with measles immunity loss at 3 and 12 months
