# Supplementary material for: Early loss of immunity against measles following allogeneic hematopoietic stem cell transplantation
Source: Am J Hematol. 2019 Aug 16;94(10):E270–2. doi: 10.1002/ajh.25590 (PMC6771529; doi:10.1002/ajh.25590)
Supplement: Supplementary file 1 — Table S1 Baseline Characteristics Table S2: Factors associated with measles immunity loss at 3 and 12 months [file AJH-94-E270-s001.docx]

**Supplementary Data Table 1: Baseline Characteristics**

|  | **N (%) / median [IQR]** |
| --- | --- |
| **Total** | 91 |
| **Male** | 55 (60) |
| **Age** | 54 [41-61] |
| **Diagnosis** |  |
| AML | 49 (54) |
| ALL | 7 (8) |
| Non-hodgkin lymfoma | 7 (8) |
| MDS | 6 (7) |
| CLL | 4 (4) |
| Hodgkin lymfoma | 4 (4) |
| Myelofibrosis | 4 (4) |
| Multiple Myeloma | 3 (3) |
| Severe aplastic anemia | 3 (3) |
| Other | 4 (4) |
| **Donor type** |  |
| MUD* | 58 (64) |
| MSD | 33 (36) |
| **Conditioning regimen** |  |
| MAC | 14 (15) |
| RIC | 77 (85) |
| **Measles immunity before HSCT** |  |
| Natural immunity measles | 72 (79) |
| Vaccine induced immunity | 13 (14) |
| No measles immunity | 6 (6.6) |
| **Acute GVHD** |  |
| Grade 0 or I (no/mild) | 45 (49) |
| Grade II-IV (severe) | 46 (51) |
| **Chronic GVHD** |  |
| None or mild | 59 (65) |
| Moderate or severe ^¶^ | 32 (35) |
| **95% Chimerism of MNCs** |  |
| 3 months post-HSCT | 81(89) |
| 12 months post HSCT | 89 (98) |
| Missing | 2 (2.2) |
| **95% Chimerism of granulocyts** |  |
| 3 months post-HSCT | 86 (95) |
| 12 months post HSCT | 89 (98) |
| Missing | 2 (2.2) |
| **IVIG use during study period^§^** | 2 (2.2) |

**Legend table 1** *All, but five of the patients with a MUD had a complete HLA-match. All patients with a HLA mismatch had a 9/10 matched donor. ¶ All cases diagnosed after day 100. ^§^ The 2 patients that used IVIG were both RIC and had natural pre-HSCT measles immunity. Both were seropositive at baseline and remained seropositive during the study period. No patients used subcutaneous immunoglobulins.

**Abbreviations**: AML= acute myeloid leukemia, ALL= acute lymphoblastic leukemia, MDS= myelodysplastic syndrome; CLL= chronic lymphoblastic leukemia; MUD= matched unrelated donor; MSD= matched sibling donor; MAC= myeloablative; RIC= reduced intensity conditioning; HSCT= hematopoietic stem cell transplantation; GvHD= graft versus host disease; MNC= mononuclear cells; IVIG = Intravenous Immunogolobulines

**Supplementary Data Table 2**: Factors associated with measles immunity loss at 3 and 12 months after HSCT

|  | **Immunity loss T3 n/total (%)** | **OR (95%CI)** | **P- value** | **Immunity loss T12 n/total (%)** | **OR (95%CI)** | **P-value** |
| --- | --- | --- | --- | --- | --- | --- |
| **Total** | 8/74 (11) | **NA** | **NA** | 33/78 (42) | NA | NA |
| **Sex** |  |  | 0.12 |  |  | 0.60 |
| Male | 4/44 (16) | ref |  | 17/46 (37) | ref |  |
| Female | 1/30 (3.3) | 0.18 (0.02-1.57) |  | 10/32 (31) | 0.78 (0.30-2.02) |  |
| **Age** |  |  | 0.17 |  |  | 0.64 |
| 18-40 y | 4/16 (25) | ref |  | 7/17 (41) | ref |  |
| 41-60 **y** | 2/36 (5.6) | 0.18 (0.03-1.09) |  | 12/37 (32) | 0.69 (0.21-2.25) |  |
| ≥61 y | 8/74 (11) | 0.30 (0.05-1.83) |  | 8/24 (33) | 0.71 (0.20-2.58) |  |
| **Donor type** |  |  | 0.95 |  |  | 0.88 |
| MUD* | 5/47 (11) | ref |  | 17/50 (34) | ref |  |
| MSD | 3/27 (11) | 0.95 (0.21-4.34) |  | 10/28 (36) | 0.93 (0.35-2.45) |  |
| **Conditioning regimen §** |  |  | **0.01** |  |  | 0.14 |
| MAC | 4/11 (36) | ref |  | 6/11 (55) | ref |  |
| RIC | 4/63 (6.3) | **0.12 (0.02-0.58)§** |  | 21/67 (31) | 0.38 (0.10-1.39)**§** |  |
| **Measles immunity before HSCT** |  |  | 0.13 |  |  | 0.58 |
| Natural immunity | 5/61 (8.2) | ref |  | 22/66 (33) | ref |  |
| Vaccine induced immunity | 3/13 (23) | 1.83 (0.83-4.04) |  | 5/12 (42) | 1.20 (0.64-2.24) |  |
| **Severe Acute GVHD (grade II-IV)** |  |  | 0.08 |  |  | 0.94 |
| Absent | 1/34 (2.9) | ref |  | 13/38 (34) | ref |  |
| Present | 7/40 (18) | 7.00 (0.82-60.1) |  | 14/40 (35) | 1.03 (0.41-2.63) |  |
| **Moderate to severe chronic GVHD^*^** |  |  |  |  |  | 0.10 |
| Absent | 8/74 (11) | NA |  | 14/50 (28) | ref |  |
| Present | 0/0 (0) | NA |  | 13/28 (46) | 2.23 (0.85-5.85) |  |
| **95% Chimerism 3 months post-HSCT** |  |  |  |  |  |  |
| **MNCs** |  |  | 0.76 |  |  | 0.19 |
| No | 1/12 (8.3) | ref |  | 3/8 (38) | ref |  |
| Yes | 7/62 (11) | 1.40 (0.16-12.5) |  | 24/70 (34) | 0.46 (0.19-0.46) |  |
| **Granulocytes** |  |  | 0.63 |  |  | 0.86 |
| No | 1/6 (17) | ref |  | 7/14 (50) | ref |  |
| Yes | 7/68 (10) | 0.57 (0.06-5.64) |  | 20/64 (31) | 0.87 (0.19-3.95) |  |

**Legend table 3:** HSCT= hematopoietic stem cell transplantation; T3= 3 months after HSCT; T12 = 1 year after HSCT; NA= not applicable; MUD= matched unrelated donor; MSD= matched sibling donor; MAC= myeloablative; RIC= reduced intensity conditioning; GVHD= graft versus host disease; MNC= mononuclear cells; * Since all cases of cGVHD in this study were diagnosed after month 3, cGVHD could not be analyzed at T3. §When adjusting ‘conditioning regimen’ for ‘age/measles immunity before HSCT/GvHD’ in multivariable regression analysis, similar odds ratios and CIs were found (Data not shown).
